# Treatment of Dilatation of the Stomach

**Published:** 1893-01-28

**Authors:** 


### THE HOSPITAL CLINIC.

[*The Editor will be glad to receive offers of co-operation and contributions from members of the profession All letters should be addressed to* THE EDITOR, THE LODGE, PORCHESTER SQUARE, LONDON, W.]

### ST. MARY'S HOSPITAL.

### TREATMENT OF DILATATION OF THE STOMACH.

The treatment of dilatation of the stomach at St. Mary's may, for the sake of convenience, be discussed under the following heads: (1) The dietetic treatment; (2) The medicinal treatment; (3) The mechanical treatment; (4) The surgical treatment.

*The Dietetic Treatment.*—It is impossible to lay down exact dietetic rules in the treatment of gastric dilatation, as it is commonly found necessary to treat each case on its merits. Dietetic treatment is, however, of the utmost importance, and it should be conducted on the following general principles: As regards quality of food, the kind of diet employed depends mainly on the condition of the gastric juice. If the gastric secretion is found to contain free hydrochloric acid, nitrogenous food may be given with a suitable quantity of fats and hydro-carbons. Should the gastric juice contain no free hydrochloric acid, and should there be evidence of considerable fermentation of the gastric contents, as shown by the presence of acetic or butyric acids, and large quantities of sarcinæ, &c., in the vomited matter, fats and hydro-carbons must be withheld, and and the patient fed chiefly on nitrogenous food. In these cases, too, the food is often most advantageously given in a peptonised form. It may be pointed out that the difference in dietetic treatment above indicated, is not of very great value from a practical point of view. In the great majority of cases it is found advisable from the outset to administer the most bland and digestible forms of food, and to prohibit as far as possible the use of all starchy and fatty compounds. Should the food given by the mouth be insufficient, or, in spite of the treatment to be presently described, should it be vomited, the patient must be fed on nutrient enemata. It is essential that the food be given in small quantities, and at short and regular intervals. In severe cases it is the custom at St. Mary's to give food every two or three hours. Rest must be enjoined for at least an hour after each meal in the case of out-patients. The amount of liquid taken is restricted as much as possible. Alcoholic liquors and all aërated waters are entirely prohibited. The main object to be attained by the dietetic treatment of gastric dilatation is to supply the largest quantity of nutritive material in the smallest possible bulk and in the least irritating form, in order that the stomach may have as much rest as possible.

*The Medicinal Treatment.*—If there is much gastric irritability and discomfort it is well to commence by giving a mixture containing bismuth, alkalies, and the tincture of nux vomica (mx-mxv) before meals. Pain may be relieved by the use of morphine or belladonna. Hydrocyanic acid is sometimes found very useful in the treatment of pain and sickness. Fermentation of the gastric contents must be checked by the administration of carbolic acid (gri.) or creosote (mi.) in the form of pill after meals. The former drug is the one most commonly employed at St. Mary's, made into a pill with mica Panis (q.s.). The hyposulphites, sulphurous acid (ʒi.-ʒiii aq.) or sulpho carbolate of soda are sometimes employed with the same object. The latter drug is a convenient one to use, as it can be given with the bismuth mixture already mentioned, in doses of from gr. x. to gr. xx., after meals, and is generally found to be most efficacious.

Pepsine, given immediately after food, is frequently found to be productive of good results, but the treatment must not be continued too long. In those cases in which hydrochloric acid is absent from the gastric juice it should be administered instead of alkalies after meals.

Later, when the gastric irritability has diminished, a mixture of nitro-muriatic acid with the tincture of nux vomica, and a stomachic given before meals is useful.

*The Mechanical Treatment.*—The most useful treatment for the relief of the symptoms caused by gastric dilatation consists in the systematic washing out of the stomach. This operation, though a simple one, and one in which tolerance is soon established, must be carried out with the utmost care and gentleness, as, in spite of every precaution, accidents have followed the use of the remedy.

The following is a brief description of the apparatus used at St. Mary's, and the mode of procedure commonly adopted: The instrument required consists of a soft, flexible, india-rubber "stomach tube," about two feet in length, connected by means of glass or vulcanite tubing with an ordinary piece of red rubber tubing, three feet long, to the free end of which is attached a vulcanite or glass funnel. The connections between the two tubes and the funnel are preferably to be made of vulcanite, as there is a possibility that the glass might break when warm water is poured over it, and consequently pieces of glass might get washed into the stomach. A jug containing eight or nine pints of the fluid with which it is intended to wash out the stomach must be at hand. This fluid may consist of warm water simply at a temperature of 100 deg. F. At St. Mary's it is the custom to use warm water alone, or a warm alkaline solution (sodæ bicarb. (gr. x.-xv.—ʒj.) or a weak solution (1.3 per cent.) of boracic acid. Various other antiseptic solutions may occasionally be used. A pail or basin to receive the stomach washings must be placed by the side of the chair or bed.

The mode of procedure is then as follows: The patient sits up in bed, and the tube, previously well lubricated with sweet oil or vaseline, is passed, slightly bent downwards, into the pharynx in the middle line. This part of the operation is done as rapidly as possible, and the patient is encouraged to perform the act of swallowing while the tube is passed into the stomach. The patient, or preferably an assistant, may then hold the tube just outside the mouth to prevent it slipping. The funnel, which is held below the level of the stomach, is then filled with the warm water or prepared solution and slowly raised, and by this means the fluid is introduced into the stomach. The funnel is then depressed until it fills, and is then again raised. This manœuvre is repeated several times, and then the funnel is depressed and inverted into the receptacle for the washings. The stomach by this means is emptied of its contents. The amount of fluid injected into the stomach should not exceed one pint.

The foregoing manipulation is repeated until the washings from the stomach come out perfectly clear. When this is the case the tube may be withdrawn. Lavage of the stomach should not be performed more than once or twice in the twenty-four hours, and should of course be done before food is given.

In those cases of dilatation of the stomach in which gastric ulcer is suspected the greatest caution must be observed in washing out the organ. Care must be taken not to introduce the flexible tube too far, and the irrigation must be conducted under as low a pressure as possible. Lavage of the stomach should not be continued for more than a few weeks at a time.

Under this heading may be mentioned the treatment of gastric dilatation by means of electricity and massage. Both the faradic and galvanic current are occasionally used. With the faradic current the two poles may be placed on different points over the abdominal walls, corresponding to the position of the stomach. If the galvanic current is used the kathode is placed over the epigastrum and the anode over the lower curvical or dorsal region of the spine.

Gentle massage of the abdomen sometimes gives good results, but it should be discontinued if the patient complains of much pain or discomfort after its employment.

*Surgical Treatment.*—If, after the foregoing measures have been tried, the symptoms remain unrelieved, and especially if vomiting and pain continue, operative procedure is called for. The operation most likely to do good is that of digital stretching of the pylorus (Loreta's operation). Gastro-enterostomy has been successful in a few cases.

